# Genotype-specific physiological and transcriptomic responses to drought stress in *Setaria italica* (an emerging model for Panicoideae grasses)

**DOI:** 10.1038/s41598-017-08854-6

**Published:** 2017-08-30

**Authors:** Sha Tang, Lin Li, Yongqiang Wang, Qiannan Chen, Wenying Zhang, Guanqing Jia, Hui Zhi, Baohua Zhao, Xianmin Diao

**Affiliations:** 1 Institute of Crop Sciences, Chinese Academy of Agricultural Sciences, 100081 Beijing, People’s Republic of China; 2 College of Life Science, Hebei Normal University, 050012 Shijiazhuang, People’s Republic of China; 3 Institute of Cotton, Hebei Academy of Agricultural and Forestry Sciences, 050030 Shijiazhuang, People’s Republic of China; 4 Institute of Dryland Agriculture, Hebei Academy of Agricultural and Forestry Sciences, 050000 Hengshui, People’s Republic of China

## Abstract

Understanding drought-tolerance mechanisms and identifying genetic dominance are important for crop improvement. *Setaria italica*, which is extremely drought-tolerant, has been regarded as a model plant for studying stress biology. Moreover, different genotypes of *S. italica* have evolved various drought-tolerance/avoidance mechanisms that should be elucidated. Physiological and transcriptomic comparisons between drought-tolerant *S. italica* cultivar ‘Yugu1’ and drought-sensitive ‘An04’ were conducted. ‘An04’ had higher yields and more efficient photosystem activities than ‘Yugu1’ under well-watered conditions, and this was accompanied by positive brassinosteroid regulatory actions. However, ‘An04’s growth advantage was severely repressed by drought, while ‘Yugu1’ maintained normal growth under a water deficiency. High-throughput sequencing suggested that the *S. italica* transcriptome was severely remodelled by genotype × environment interactions. Expression profiles of genes related to phytohormone metabolism and signalling, transcription factors, detoxification, and other stress-related proteins were characterised, revealing genotype-dependent and -independent drought responses in different *S. italica* genotypes. Combining our data with drought-tolerance-related QTLs, we identified 20 candidate genes that contributed to germination and early seedling’ drought tolerance in *S. italica*. Our analysis provides a comprehensive picture of how different *S. italica* genotypes respond to drought, and may be used for the genetic improvement of drought tolerance in Poaceae crops.

## Introduction

Drought is a major constraint on crop productivity and influences ecosystems worldwide^1^. The longer and more severe drought events predicted to result from global climate change will exacerbate their negative impacts on crops and compromise food security^2^. During the long natural selection process, plants have evolved various drought-avoidance and -tolerance mechanisms that enable them to maintain growth and reproduction during insufficient water periods^3^. Understanding the morphological, physiological and transcriptional aspects of drought responses in plants is, therefore, of cardinal significance and will be useful in breeding drought-tolerant crops.

Panicoideae is a tribe of the Poaceae, which contains many important cereal and biofuel crops, such as *Zea mays*, *Sorghum bicolor* and *Panicum virgatum*. As members of the Panicoideae subfamily, *Setaria italica* (foxtail millet) and its wild ancestor *Setaria viridis*, are novel model species for understanding basic biological processes. *S. italica* has a small diploid genome (~510 Mb), short life cycle (6 weeks for some accessions), is small in stature, and undergoes self-pollination and C_4_ photosynthesis^4^. Additionally, foxtail millet has adapted well to arid and semi-arid regions during its long domestication and improvement process. Most of the foxtail millet varieties are considered to be drought-tolerant and have higher water-use efficiency values when compared with other crops, such as wheat, maize and sorghum^5^. *S. italica* is considered a model for studying the molecular mechanisms of drought tolerance in cereals^6^. Thus, identifying key genes and understanding their roles in response to drought in *Setaria* will provide insights into the drought-related molecular mechanisms of millet crops and also enable the application of this knowledge to other major crops, especially those of the Panicoideae family.

Several genome-wide studies have focused on the identification of important genetic determinants of drought tolerance in *Setaria*. Early research that concentrated on the transcriptomic responses to abiotic stress in foxtail millet used subtractive hybridisation analyses and cDNA-AFLP methods, but the research suffered from the lack of a complete reference genome and annotation databases^7–9^. With the release of the entire *S. italica* genome and the development of next-generation sequencing (NGS) technologies^10^, a few reports are now available on the identification of stress-responsive genes and microRNAs (miRNAs) involved in the drought tolerance of *S. italica*. Qi *et al*. (2013) analyzed the transcriptome of foxtail millet under polyethylene glycol (PEG)-induced drought conditions and identified 2,824 genes and 215 miRNAs^11^. Yadav *et al*.^12^ subjected two foxtail millet cultivars to a 20% PEG-6000-induced dehydration stress, and identified 55 known and 136 novel miRNAs using Illumina sequencing technology^12^.

Previous research provided useful information for studying drought-responsive genes and miRNAs in foxtail millet. However, most of these studies employed PEG-simulated drought stress, which is essentially different from natural soil drought events. In addition, previous studies indicated that different genotypes of the same species showed different regulatory responses under stress, and more genes were differentially expressed in the sensitive cultivar than in the tolerant cultivar^13^. In this study, we screened two *S. italica* genotypes having contrasting drought-tolerance levels from *S. italica* core germplasms^14^. Seedlings of these two foxtail millet genotypes were subjected to a natural drought treatment. Physiological and biochemical tests indicated that these two genotypes have different drought-response behaviors. Using NGS transcriptome sequencing, a core set of drought-regulated genes was determined, as well as special biological functions and metabolic pathways. An associated analysis of these genes with previously mapped quantitative trait loci (QTLs)^15^, identified several important candidate genes that contribute to early seedling’ drought tolerance in *Setaria*, and they may serve as reliable gene resources for studying drought tolerance in crops.

## Results and Discussion

### Phenotypic and physiological analyses identified two *S. italica* cultivars exhibiting contrasting drought tolerance levels

**Figure 1:**
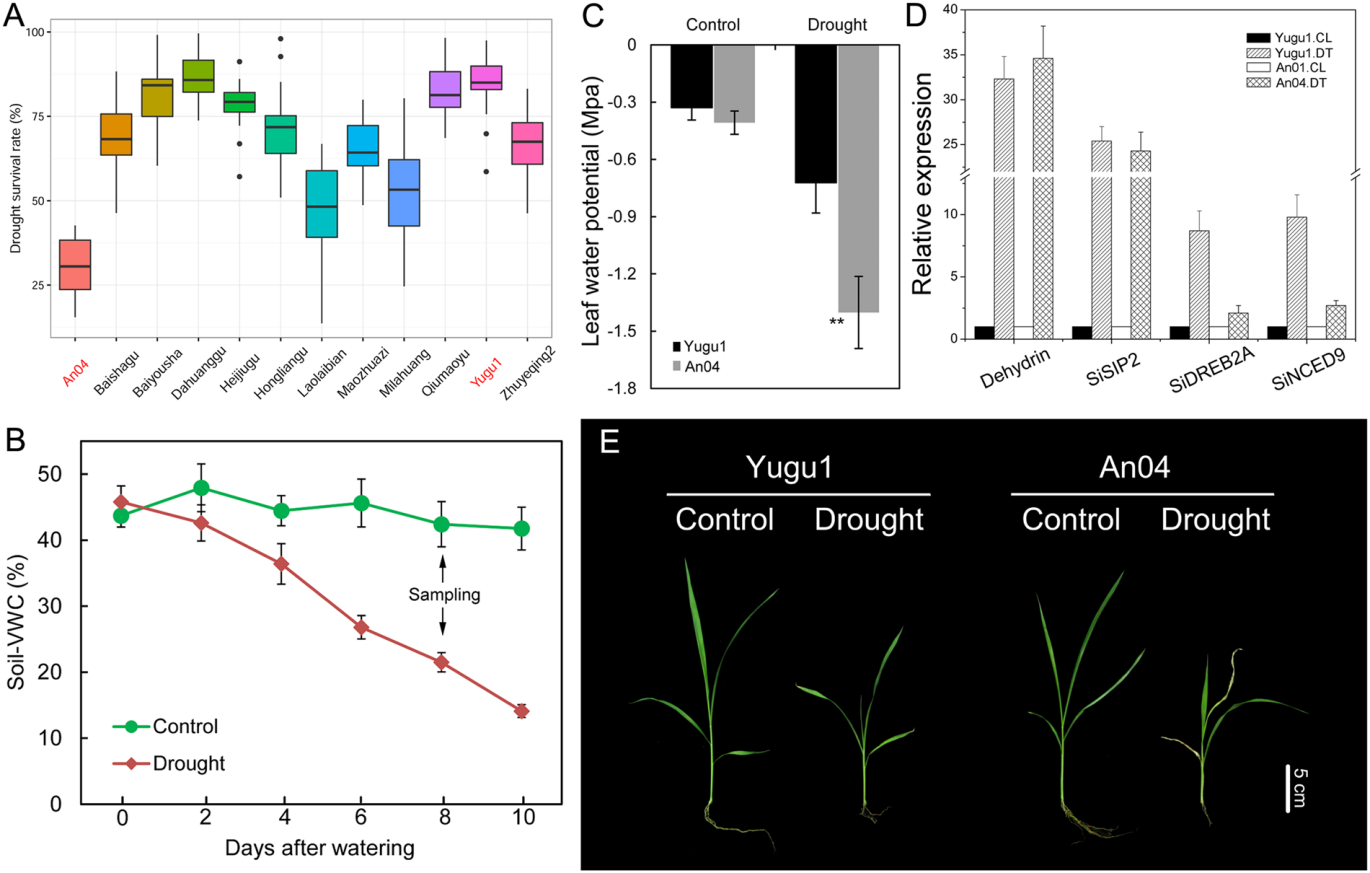
Effects of drought stress on different foxtail millet genotypes. (**A**) Drought survival rates of 12 foxtail millet landraces in the drought/rewatering experiment. The phenotypic data were obtained from six replicated experiments, and each experiment contained 15 biological replicates. The experimental procedure follows that of a previous study^16^. (**B**) Soil volumetric water content during the 10-day drought treatment. (**C**) The leaf water potential (LWP) of ‘An04’ plants decreased to approximately onefold lower than that of ‘Yugu1’ after 8 d of drought. The arrow indicates the day on which we sampled the plant materials for RNA-seq. (**D**) The expression levels of four representative drought-responsive genes from ‘Yugu1’ and ‘An04’ plants were analyzed by qPCR. The four drought-inducible marker genes were a dehydrin (Seita.8G115400), *SiDREB2A* (Gene ID: Seita.4G016400), *SiSIP2* (Seita.2G081000), and *SiNCED9* (Seita.9G156500). Error bars represent standard errors of the mean (n = 6). (**E**) Phenotypic alterations of ‘Yugu1’ and ‘An04’ in response to drought.

A significant difference in seedling survival rates between ‘Yugu1’ and ‘An04’ was detected (Fig. 1A) by screening the responses of 500 diverse foxtail millet varieties (selected from our previous study^14^) to water deficiency. The large-scale drought/rehydration screening experiment was conducted according to a previous report on maize^16^. For physiological measurements, healthy and uniformly developed ‘Yugu1’ and ‘An04’ seedlings were exposed to a gradually increasing soil water depletion. As shown in Fig. 1B, the soil volumetric water content declined accordingly, falling below 21.3% in the drought treatment on the 8th d, while remaining steady in controls (~42.4%). The leaf water potential (LWP) of ‘Yugu1’ and ‘An04’ seedlings were monitored. The LWP of ‘An04’ plants decreased to −2.5 ± 0.37 MPa on the 8th day of water withholding, which was approximately onefold lower than the LWP of ‘Yugu1’ (Fig. 1C, P < 0.001). At this time point, we sampled drought-stressed ‘An04’ and ‘Yugu1’ plants, as well as their well-watered controls, to construct high-throughput sequencing libraries. Concurrently, these samples were used for the qRT-PCR analysis of four drought-inducible genes, *SiDREB2A* (Gene ID: Seita.4G016400), *SiSIP2* (Seita.2G081000), *SiNCED9* (Seita.9G156500) and a dehydrin (Seita.8G115400)^10^. qPCR results validated the drought treatment and sampling methods (Fig. 1D). The phenotypic changes of these two cultivars in response to drought are illustrated in Fig. 1E. ‘Yugu1’ maintained relatively better growth after water was withheld for 8 days, while the leaves of ‘An04’ were beginning to wilt. Based on their phenotypic and physiological differences during drought responses, ‘Yugu1’ was defined as a drought-tolerant variety, while ‘An04’ was defined as a drought-sensitive variety.

**Figure 2:**
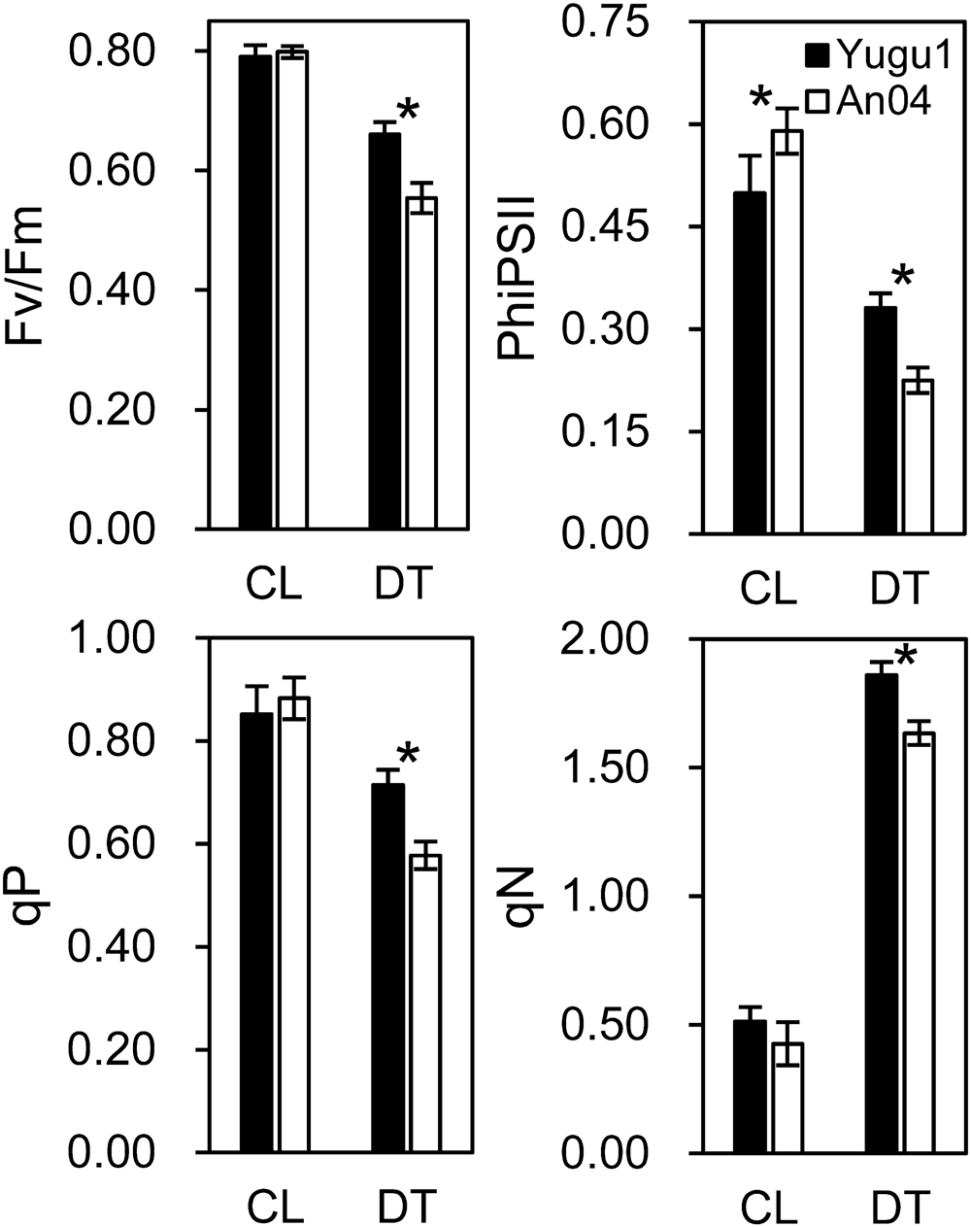
Chlorophyll fluorescence in ‘Yugu1’ and ‘An04’ plants. Each condition includes measurements of 18 biological replications. CL, well-watered control; DT, drought treatment; Fv/Fm, maximum energy conversion efficiency in PSII centers; ΦPSII, quantum yield of photosystem II electron transport; qP, photochemical quenching coefficient; qN, non-photochemical quenching coefficient; Asterisks indicate a significant difference between ‘Yugu1’ and ‘An04’ plants: n = 18, Welch’s two-sample t-test, P < 0.001.

Chlorophyll fluorescence, as indicated by the Fv/Fm ratio, positively correlates with drought-stress responses^17^. The Fv/Fm ratio significantly declined in both foxtail millet varieties under drought conditions, while a greater reduction occurred in the drought-sensitive plants (‘An04’) compared with ‘Yugu1’ (Fig. 2 and Supplementary Table S1). Similar trends were found for quantum yield of PSII electron transport (ΦPSII) and photochemical quenching (qP). In addition, non-photochemical quenching sharply increased in drought-stressed ‘Yugu1’ and ‘An04’ (~3.62 and ~3.83 fold, respectively), indicating that when foxtail millet was subjected to a water deficiency, more energy was consumed as heat rather than being used in carbohydrate assimilation. The changes in chlorophyll fluorescence suggested that the functions of Photosystem II were repressed in both ‘Yugu1’ and ‘An04’ varieties during stress, even though ‘Yugu1’ maintained a relatively higher light-use efficiency than ‘An04’ under drought conditions.

### Genome-wide identification of differentially expressed genes in the two foxtail millet genotypes in response to drought

**Figure 3:**
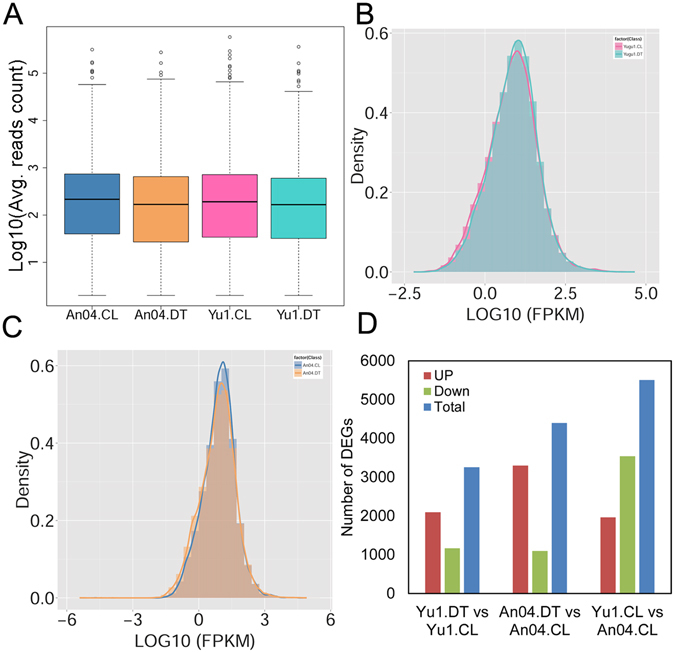
Analyses of gene expression levels in *S. italica* subjected to drought stress. (**A**) Box plots show the range of read counts mapped to the expressed genes in the four samples. The read count values are averages of three biological replications. (**B**,**C**) Distribution of gene expression (estimated by FPKM) in ‘Yugu1’ (**B**) and ‘An04’ (**C**) under control and drought conditions, respectively. (**D**) Number of differentially expressed genes in each comparison group.

To achieve a better understanding of drought-tolerance mechanisms in foxtail millet, we employed RNA-seq to analyze the transcriptomes of ‘Yugu1’ and ‘An04’ under control (named as Yu1.CL and An04.CL, respectively) and drought (Yu1.DT and An04.DT, respectively) conditions. Because the reliability of the RNA-seq data analysis is of fundamental importance for discovering biologically important changes in gene expression, and normalization is an important consideration^18^, we used three mainstream computational packages (Cufflinks^19^, edgeR^20^, and DESeq. 2^21^) to measure mRNA expression levels. Only genes showing concordant expression levels in at least two packages were identified as reliable differentially expressed genes (DEGs). In summary, expression data were available for 27,667 (78.0%) genes of the foxtail millet reference genome. The number of reads mapped to different genes was broad and ranged from 1 to ~0.36 million, with a median of 391 for all of the expressed genes (Fig. 3A). The densities of the expression levels for the detected genes formed a standard normal distribution, and only a small portion of genes were weakly or highly expressed (Fig. 3B and C). In total, 9,652 DEGs were identified with estimated absolute log_2_ fold changes ≥1 and false discovery rates ≤ 0.01. The distribution of these DEGs among different groups is shown in Fig. 3D. qRT-PCR was conducted to validate the DEG data (Supplementary Table S2). We found that 31 out of 34 (91.2%) genes showed concordant expression trends between qPCR and RNA-seq. A statistical analysis showed a correlation coefficient (r) of 0.775, indicating that the DEG data was generally reliable (Supplementary Table S3). The expression values and annotations of all of the DEGs are listed in Supplementary Table S4.

### K-means clustering and functional enrichment analysis of DEGs

**Figure 4:**
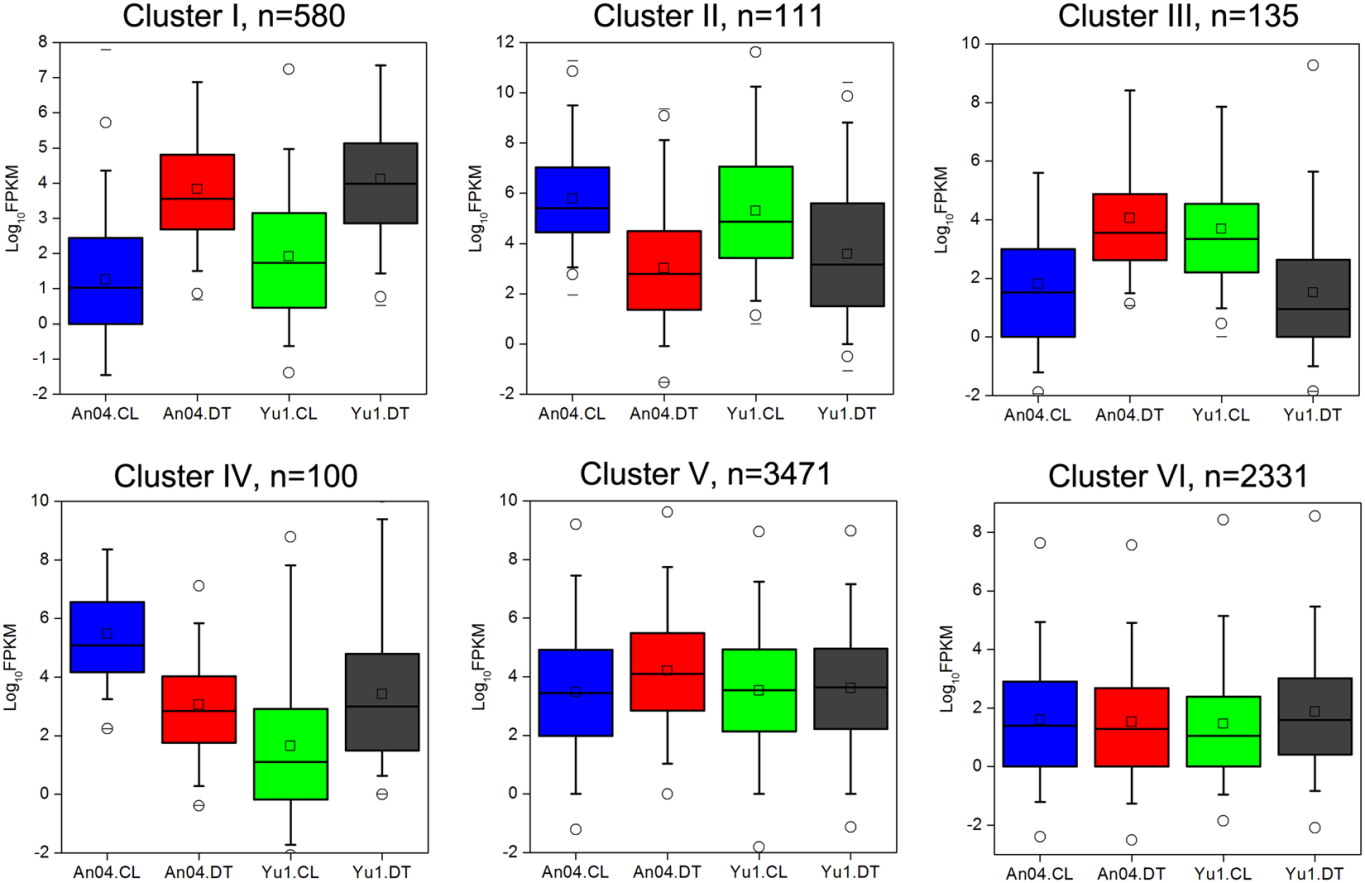
K-means clustering of gene expression features in ‘Yugu1’ and ‘An04’ in response to drought stress. *n* represents the number of differentially expressed genes found in each cluster. The y-axis represents normalized gene expression values.

To analyze the regulatory processes that are critical for foxtail millet drought tolerance, DEGs were grouped by their expression signatures across the four samples (Yu1.CL, Yu1.DT, An04.CL, and An04.DT). The k-means clustering analysis suggested that the majority of the genes could be divided into six clusters (Fig. 4).

The GO enrichment analysis identified biological processes that were regulated at the transcriptional level in each of the six clusters (Supplementary Table S5). Cluster I contained the 580 genes that were up-regulated in both varieties during drought. The drought-stressed ‘Yugu1’ and ‘An04’ seedlings contained high numbers of transcripts involved in stress and defense responses (e.g., GO:0006952, GO:0009415 and GO:0009628), abscisic acid (ABA) signaling (e.g., GO:0009737 and GO:0009738), and homeostasis maintenance (e.g., GO:0055114 and GO:0006026). Cluster II included the 111 genes that were more abundant under well-watered conditions in both varieties. In this cluster, photosynthetic light-harvesting and metabolism processes related to lipid, amino acid and carbohydrate biosynthesis were enriched, indicating that these processes are important for plant normal growth, but they were inhibited in response to drought. Cluster III contained the 135 genes that were up-regulated in the drought-sensitive variety (An04) but down-regulated in the drought-tolerant plant (Yugu1) when confronted with a water deficiency. Interestingly, genes involved in energy metabolism, such as photosynthesis, ATP formation and cellular respiration, were enhanced in ‘An04’ but repressed in ‘Yugu1’. The drought-tolerant cultivar may inhibit the above processes to protect the plant’s cells from excessive electrons and reactive oxygen species (ROS) production. Cluster IV contained 100 genes that had opposite expression patterns compared with those of Cluster III. Abundant genes related to cell division, cell cycle and cell proliferation were up-regulated in the drought-tolerant variety, but repressed in the drought-sensitive ‘An04’ plants. This suggested that ‘Yugu1’ could maintain cell growth under water stress, while the growth of ‘An04’ was severely affected. These results corroborated our phenotypic and physiological analyses (Figs 1 and 2). The larger Clusters V and VI had 3,471 and 2,331 genes, respectively, that were only differentially expressed in ‘An04’ or ‘Yugu1’ varieties in response to drought. The DEGs enriched only in ‘An04’ (Cluster V) mainly represented biological functions related to cellular macromolecular/component biogenesis (e.g., GO:0044085 and GO:0009832) and organization (e.g., GO:0006325 and GO:0006996). The drought-stressed ‘Yugu1’ sample contained a number of genes that participated in protective product biosynthesis (e.g., GO:0009699, GO:0030418 and GO:0016102) and defense (e.g., GO:0006979 and GO:0009737).

Metabolic pathways involved in drought-stress responses in foxtail millet were delineated through the KEGG system. As shown in Supplementary Table S6, a number of pathways that were conducive to *S. italica* drought adaptation were enriched in both drought-tolerant and -sensitive genotypes, including plant hormone signal transduction, MAPK signaling pathway, phenylpropanoid biosynthesis, galactose metabolism, tyrosine metabolism, and glutathione metabolism. However, genes involved in biomass accumulation, such as starch and sucrose metabolism, fatty acid elongation, and photosynthesis pathways were inhibited by drought.

### An04 genotype produces greater yields than Yugu1 under well-watered conditions accompanied by positive brassinosteroid (BR) regulations at the transcriptome level

**Figure 5:**
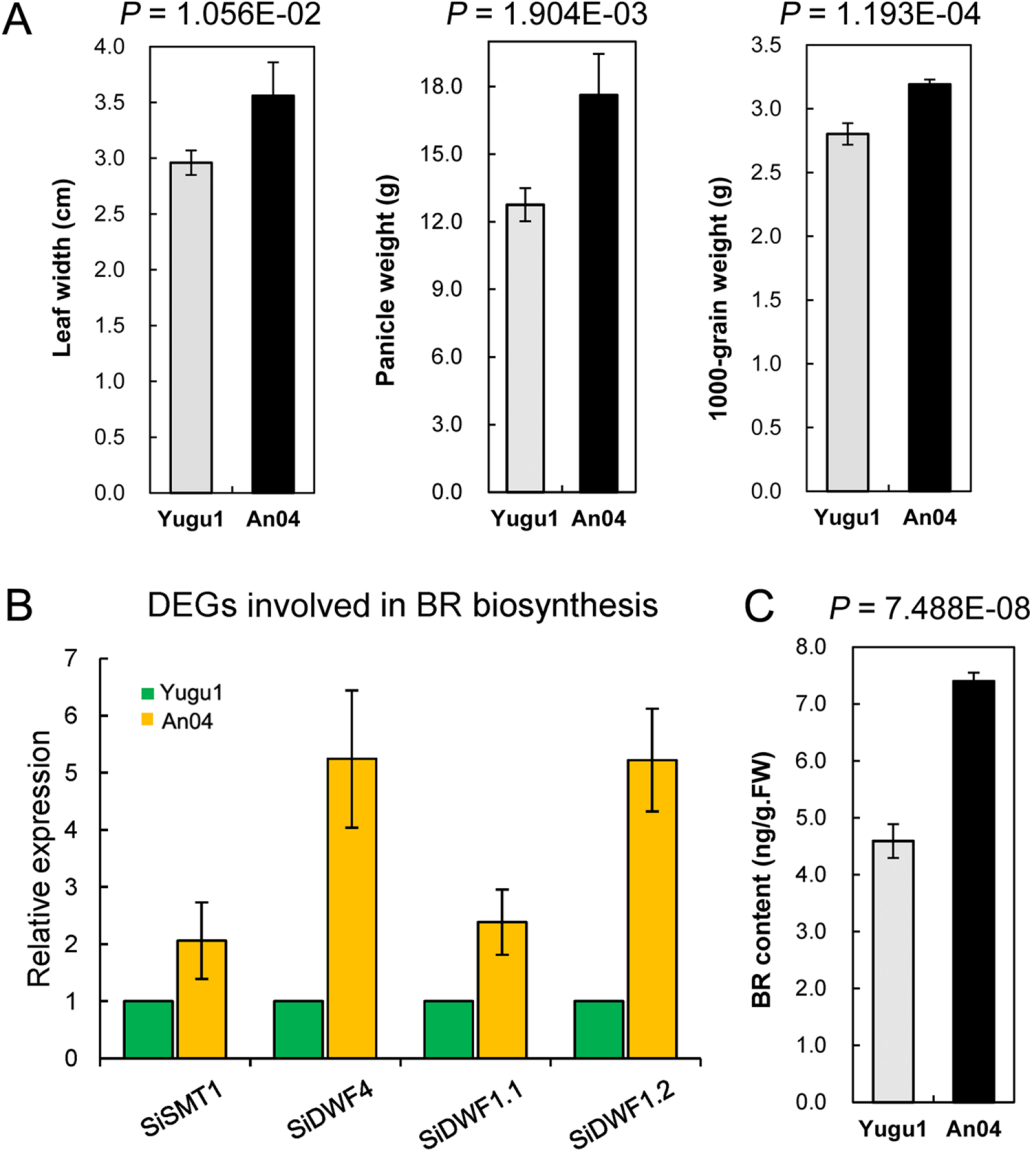
Growth differences of ‘Yugu1’ and ‘An04’ plants under well-watered conditions. (**A**) Comparison of agronomic traits between ‘An04’ and ‘Yugu1’. Five individual plants per cultivar were measured for each agronomic trait. (**B**) Comparisons of expression levels of key regulatory genes in brassinosteroid (BR) biosynthesis between ‘An04’ and ‘Yugu1’ using qPCR. Four BR marker genes, *SiSMT1* (Gene ID: Seita.2G079200), *SiDWF4* (Seita.7G132100), *SiDWF1.1* (Seita.4G289300) and *SiDWF1.2* (Seita.9G429800) were used in the qPCR experiment. Error bars represent standard errors of the mean (n = 6). (**C**) BR content in ‘Yugu1’ and ‘An04’ plants. Seedlings from Yugu1.CL and An04.CL groups were used for BR measurements. The data presented are the means of six replications. P values were calculated using Welch’s two-sample *t*-test.

The growth of ‘An04’ was severely repressed by drought when compared with that of ‘Yugu1’ (Fig. 1). To investigate whether this difference occurred during normal growth and development, we compared major agronomic traits between ‘An04’ and ‘Yugu1’ under well-watered conditions (Fig. 5A and Supplementary Table S7). In contrast to the growth inhibition under drought stress, ‘An04’ individuals exhibited wider leaves and greater biomasses at the seedling stage compared with ‘Yugu1’ seedlings. More remarkably, ‘An04’ produced significant greater crop grain yields (including a greater panicle weight, 1000-grain weight, and grain weight per plant) than those of ‘Yugu1’. At the transcriptomic level, we identified 5,505 genes that were differentially expressed between ‘An04’ and ‘Yugu1’ under well-watered conditions. A functional enrichment analysis showed that genes involved in cell division (P = 7.05E-37), cell wall organization or biogenesis (P = 3.43E-31), and organelle organization (1.27E-26) were more highly transcribed in ‘An04’ compared with in ‘Yugu1’. The BR metabolism pathway was especially enriched in ‘An04’. Four genes that participate in BR biosynthesis were up-regulated in ‘An04’ compared with in ‘Yugu1’ (Fig. 5B). We also measured the phytohormone content and confirmed that ‘An04’ had a significantly greater endogenous BR accumulation than ‘Yugu1’ (Fig. 5C). Of these BR-related DEGs, *SiDWF4* (Gene ID: Seita.7G132100), which was up-regulated 5.3-fold in ‘An04’ compared with in ‘Yugu1’, encodes a 22α hydroxylase and is a rate-limiting enzyme in the BR biosynthetic pathway^22^. Its homologous gene *AtDWF4* regulates leaf growth by promoting cell expansion in *Arabidopsis* ^23^, and the overexpression of *DWF4* in *Brassica napus* can increase seed yields^24^. Thus, these results may explain the wider leaves and better grain yields of ‘An04’ at the transcriptomic level.

### Foxtail millet transcriptomes were remodeled by genotype × drought stress interactions

**Figure 6:**
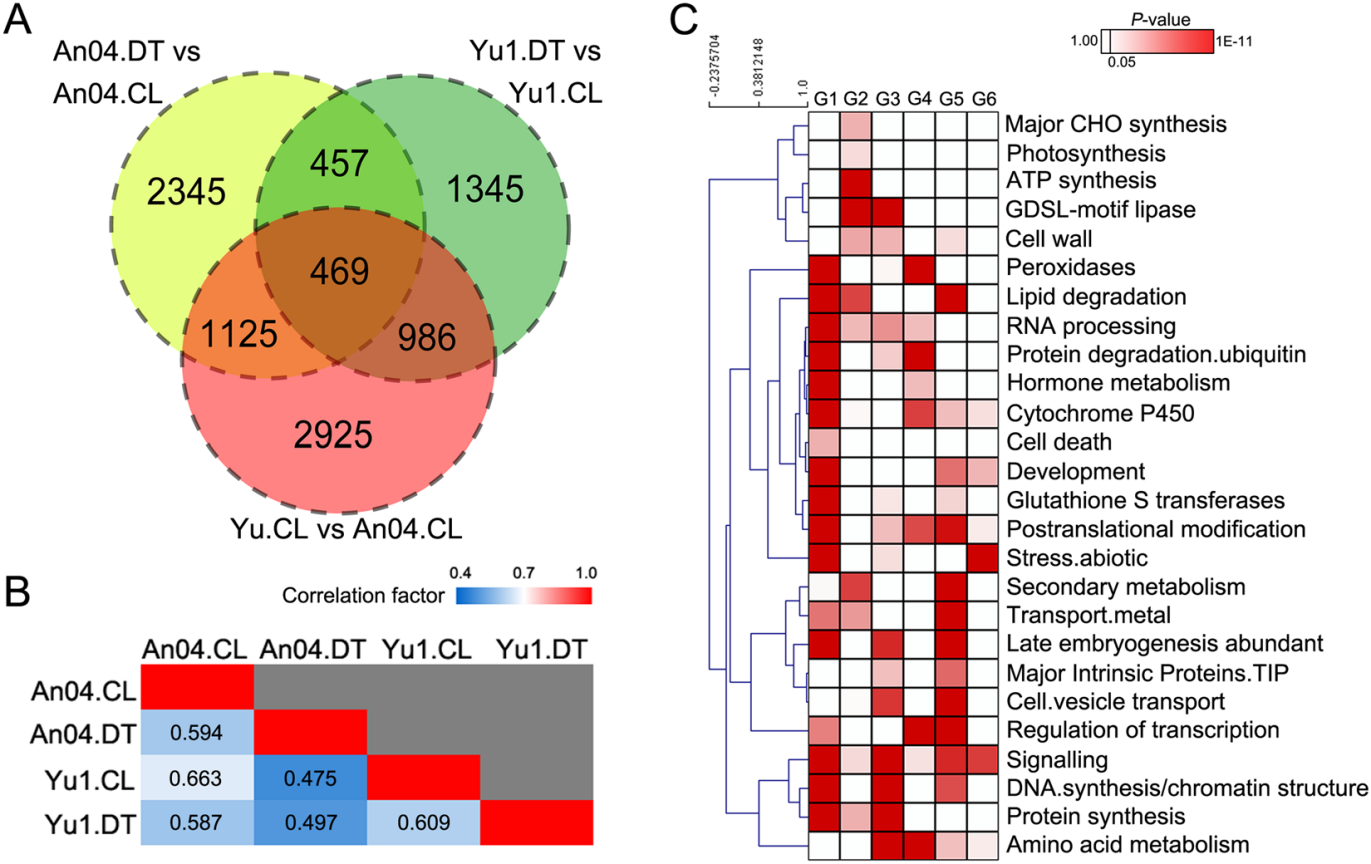
The dynamic progression of the *Setaria* transcriptome was affected by genotype and drought stress. (**A**) A Venn diagram of the numbers of differentially expressed genes in different comparisons among groups. (**B**) Correlation analysis of the expressed gene pairs among different groups. The color bar from red to blue represents the highest (1.0) to lowest (0.4), respectively, correlation values. (**C**) Functional category (modified MapMan BINs) enrichment levels among different group comparisons. The red color gradation indicates significantly enriched functional categories (P ≤ 0.05). White blocks represent MapMan BINs that were not significantly enriched. G1–G6 represent up-regulated (G1)/down-regulated (G2) genes in An04.DT *vs* An04.CL, down-regulated (G3)/up-regulated (G4) genes in Yugu1.CL *vs* An04.CL, and up-regulated (G5)/down-regulated (G6) genes in Yugu1.DT *vs* Yugu1.CL.

Comparisons of genes and their expression patterns between the drought-tolerant and -sensitive genotype of *S. italica* in response to the same level of water stress aid in the understanding of regulatory mechanisms associated with drought and also in identifying the roles of independent genotypic backgrounds in the regulation. Of these DEGs, 5,505 genes were differentially expressed between the two different genotypes under control conditions (Yugu1.CL *vs* An04.CL), while 4,396 (An04.DT *vs* An04.CL) and 3,257 (Yugu1.DT *vs* Yugu1.CL) genes were differentially expressed in drought-sensitive and -tolerant *S. italica* cultivars, respectively, in response to a water-deficiency (Fig. 6A). We did not compare An04.DT to Yu1.DT because two variables existed between these two groups. Both drought and genetic background affect gene expression levels in An04.DT *vs* Yu1.DT. In the combined drought-responsive and genotype-specific DEG data set, more than 68.5% (6,615) of the genes were unique to only one data set, 26.6% (2,568) of the DEGs were shared by two data sets, and only ~4.8% (469) were differentially expressed in all three data sets (Fig. 6A). A GO enrichment analysis was carried out for each category of genes in Fig. 6A. The results provide biological explanations for each category (Supplementary Table S8). For example, 2,925 genes were unique in the Yugu1.CL *vs* An04.CL group (Fig. 6A). This category contains genes that play important roles in regulating genotype-specific growth performance in foxtail millet, but they do not participate in drought responses. The GO enrichment analysis indicated that these genes were mainly involved in basic developmental and cell metabolism processes, such as ‘DNA replication’, ‘cell wall organization or biogenesis’, and ‘floral organ development’ (Supplementary Table S8). For the 1,345 DEGs that were only included in drought-stressed ‘Yugu1’, the GO terms related to stimulus responses and secondary metabolic processes were especially enriched, suggesting that the drought-tolerant variety is apt to activate stress-responsive genes under drought conditions (Supplementary Table S8). The 457 DEGs shared by An04.DT *vs* An04.CL and Yu1.DT *vs* Yu1.CL were induced by drought in both drought-tolerant and -sensitive genotypes. The GO enrichment analysis showed that these consensus sets of drought-responsive genes across the two genotypes were abundant for various stress-response processes (Supplementary Table S8), indicating that these genes may play conserved roles across genotypes when confronting a water deficiency.

A correlation analysis among different samples can determine how much foxtail millet plants are affected by genotype and water stress. The Pearson’s correlation coefficients (PCCs) between any two libraries were less than 0.7 (Fig. 6B), indicating weak positive associations of gene expression with different genotypes or with the drought treatment. The cutoff value was chosen according to previous reports^25, 26^. The result also suggested that genotypes and environmental factors could have large effects on the gene expression patterns in *S. italica*. To be specific, the highest correlation existed in genes that expressed under control conditions for the two genotypes (PCC = 0.663, Yu1.CL *vs* An04.CL). Expression profile data of genes responding to drought in ‘Yugu1’ (PCC = 0.609, Yu1.DT *vs* Yu1.CL) or ‘An04’ (PCC = 0.594, An04.DT *vs* An04.CL) showed relatively lower correlations. This suggested that the environmental factor (drought) had greater effects on the gene expression pattern than the genotype. Remarkably, the lowest PCC value (<0.5) was observed when both genotype and environmental factor changed, suggesting that the genotype × environment interaction (G × E) is crucial for transcriptome remodeling. Taken together, the effects of drought stress and genetic background on the gene expression profile of *S. italica* can be ordered as follows: genotype × environment interactions >environmental stress >genotype (Fig. 6A and B).

To further determine the biological functions/genes that were regulated by water stress, dependent or independent of the genotype, all of the DEGs were assigned to functional categories using the MapMan annotation system. As shown in Fig. 6C, biological functions associated with drought responses, including ion transport, DNA synthesis, transcription factors (TFs), secondary metabolism, and vesicle transport, and a number of protective proteins, including late embryogenesis abundant (LEA) proteins, and major intrinsic proteins, were enriched in both genotypes. Genes that participated in major carbohydrate synthesis, photosynthesis, ATP synthesis, and protein synthesis are particularly enriched in the drought-sensitive genotype. It is worth noting that genes regulating cell death-associated processes were only enriched in the drought-treated ‘An04’ cultivar, indicating that water stress seriously restricted organismal growth by triggering cell death in this drought-susceptible genotype. Interestingly, a few genes encoding enzymes involved in the antioxidant system, hormone metabolism, and ubiquitin-mediated protein degradation, which are important drought-responsive pathways, were more abundant in ‘Yugu1’ compared with ‘An04’ under well-watered conditions. When subjected to drought, these genes were significantly up-regulated in drought-sensitive ‘An04’, but were steadily expressed in drought-tolerant ‘Yugu1’.

### Phytohormone metabolism and signaling are positively regulated in foxtail millet in response to drought

**Figure 7:**
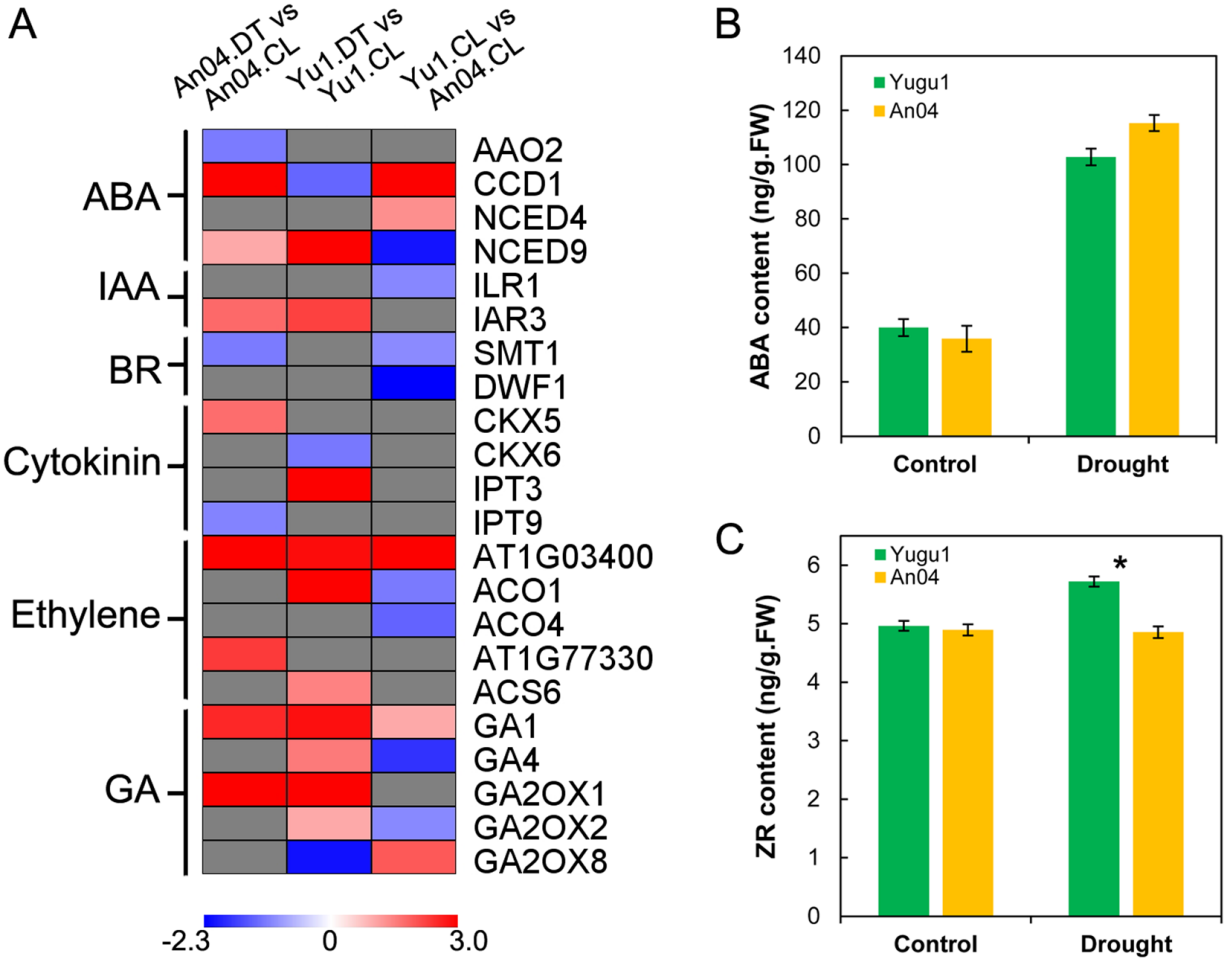
Regulation of phytohormone metabolism in different *S. italica* genotypes. (**A**) The heat map shows the expression of some featured genes involved in phytohormone metabolism. Detailed information on the differentially expressed genes involved in phytohormone metabolism and signaling can be found in Supplementary Table S9. (**B**,**C**) Abscisic acid and zeatin riboside contents in ‘Yugu1’ and ‘An04’ plants. ‘Yugu1’ and ‘An04’ seedlings were used for plant hormone measurements. The data presented are the means of six replications. Asterisks indicate a significant difference at P < 0.001.

The profiles of significantly enriched DEGs in both drought-treated ‘Yugu1’ and ‘An04’ indicated predominant roles of proteins involved in plant hormone metabolism and signaling processes. This result corroborated those of previous studies that determined that phytohormone-related processes had crucial roles in regulating plant growth and stress responses^27^. Figure 7A and Supplementary Table S9 illustrate that plant hormone metabolism-related DEGs are involved in the biosynthesis/degradation of ABA, cytokinin, gibberellin (GA), and ethylene, which appear to be transcriptionally regulated. The 9-cis-epoxycarotenoid dioxygenase (*NCED*) family of genes play essential roles in the biosynthesis of ABA^28^. Four genes from the *NCED* family were differentially expressed in drought-stressed foxtail millet. Among them, *NCED9*, which encodes a key enzyme in the biosynthesis of ABA^28, 29^, was significantly up-regulated in ‘An04’ and ‘Yugu1’, suggesting that ABA biosynthesis may be enhanced in both drought-sensitive and -tolerant genotypes. Hormone quantification experiments also verified this conclusion. The endogenous ABA level increased ~2.57-fold in both ‘Yugu1’ and ‘An04’ under drought conditions (Fig. 7B). Cytokinins regulate cell division and development, and play crucial roles in plant growth. It is worth noting that the regulation of cytokinin metabolism was genotypically different in *S. italica*. Genes that participated in the cytokinin biosynthetic process (*IPT3* and *IPT9*) were strongly up-regulated in the drought-tolerant cultivar under stress, but showed no significant increase, or were even repressed, in drought-sensitive ‘An04’. Meanwhile, CKX5 and CKX6, which catalyze the degradation of cytokinins, were abundant in the drought-sensitive cultivar, but were suppressed in drought-tolerant ‘Yugu1’ (Fig. 7A). The zeatin riboside (ZR) level was significantly higher in ‘Yugu1’ than in ‘An04’ under drought conditions. The expression levels of genes involved in the cytokinin metabolism pathway also supported these results (Fig. 7A and C). The dynamic cytokinin regulatory patterns in different *S. italica* genotypes in response to drought strongly indicated that the drought-tolerant cultivar accumulated more cytokinin to maintain growth, while the drought-sensitive cultivar was more apt to reduce the endogenous cytokinin content, which may further inhibit cell division and growth in plants subjected to a water deficiency.

In total, 54 DEGs encoding proteins involved in ABA, auxin, cytokinin, GA, ethylene, and BR signal transduction were identified. As shown in Supplementary Tables S9 and 22 genes that participated in auxin signaling and 16 that participated in ABA signal transduction, were responsive to drought. These constituted the majority of (70.4%) the detected phytohormone signaling genes, suggesting the predominant role of ABA and auxin signaling in the drought responses of foxtail millet. Additionally, some key regulators in the other three plant hormone signaling pathways were also transcriptionally regulated in response to drought in *S. italica*, including *GID1* in GA signaling, AHP histidine phosphotransfer proteins in cytokinin signaling, and *BSK1*/*BZR1* in BR signaling.

### Changing inventories of drought-responsive TFs in different foxtail millet genotypes

TFs can mediate plant growth and drought-stress responses by regulating various stress-inducible genes^30^. In total, 556 differentially expressed TFs were detected in ‘Yugu1’ or ‘An04’ under drought conditions, and these were mainly distributed into 25 TF families. TFs belonging to AP2/EREBP, C2H2/C3H zinc, MYB, bHLH, NAC, and WRKY were the most abundant in both ‘Yugu1’ and ‘An04’ accessions and accounted for over 70% of all of the differentially expressed TFs (Supplementary Table S10-TF category). As expected, these TF families have common functions in drought-stress regulation^31^.

**Figure 8:**
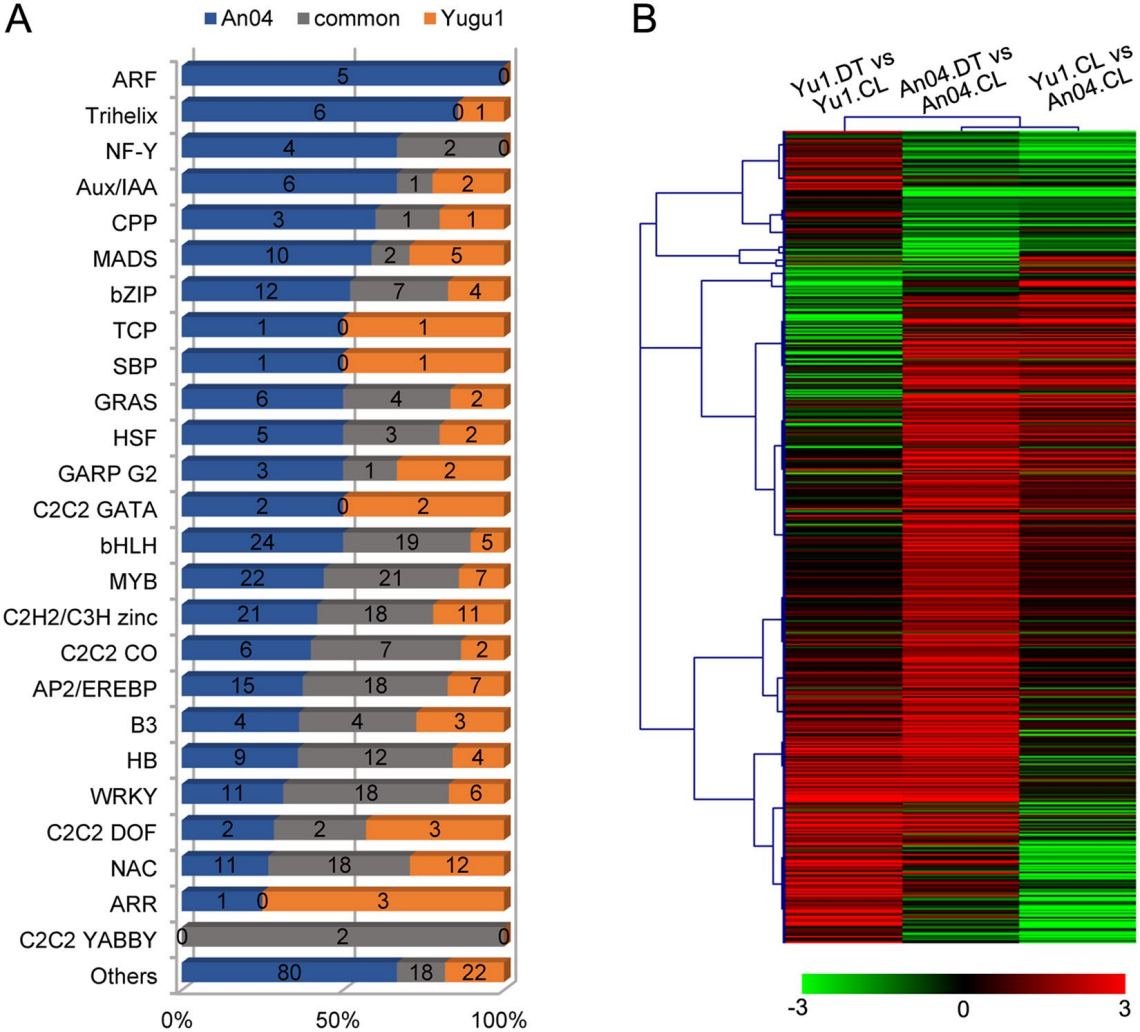
Changes in transcription factor (TF) expression in different *S. italica* genotypes. (**A**) Distribution of TF families in ‘Yugu1’ and ‘An04’ in response to drought. Blue and orange bars indicate TFs only differentially expressed in ‘An04’ and ‘Yugu1’, respectively. The grey bar shows TFs differentially expressed in both genotypes. The *y*-axis represents the percentage of the total gene number. (**B**) The heat map shows the expression profiles of the differentially expressed TFs in comparisons among different groups.

We also identified genotype-specific expression trends for TFs. Members of the ARF, Trihelix, NF-Y, AUX/IAA, CPP and MADS TF families were mainly differentially expressed in the drought-sensitive cultivar (Fig. 8A). These gene families are important transcriptional regulators involved in auxin response^32^, cell growth^33^, and flower development^34, 35^. In the drought-tolerant ‘Yugu1’, the most abundant TFs were mainly distributed in the NAC, C2C2 DOF, and WRKY families, which are major players in water-stress responses^30^. The expression pattern and hierarchical clustering analysis of all 556 differentially expressed TFs were illustrated in Fig. 8B and Supplementary Table S10-TF list. Only 25.0% of these have consistent expression patterns in both drought-tolerant and -sensitive genotypes. The majority of differentially expressed TFs (418) were under genotype-specific regulation. Interestingly, TF gene expression patterns in ‘Yugu1’ under well-watered conditions (compared with well-watered ‘An04’) were correlated to those in drought-stressed ‘An04’ (compared with well-watered ‘An04’), suggesting that TFs related to drought defense/responses were more abundant in the drought-tolerant genotype even in a normal growth environment.

### Protective genes play important roles in *S. italica’*s drought acclimation process

**Figure 9:**
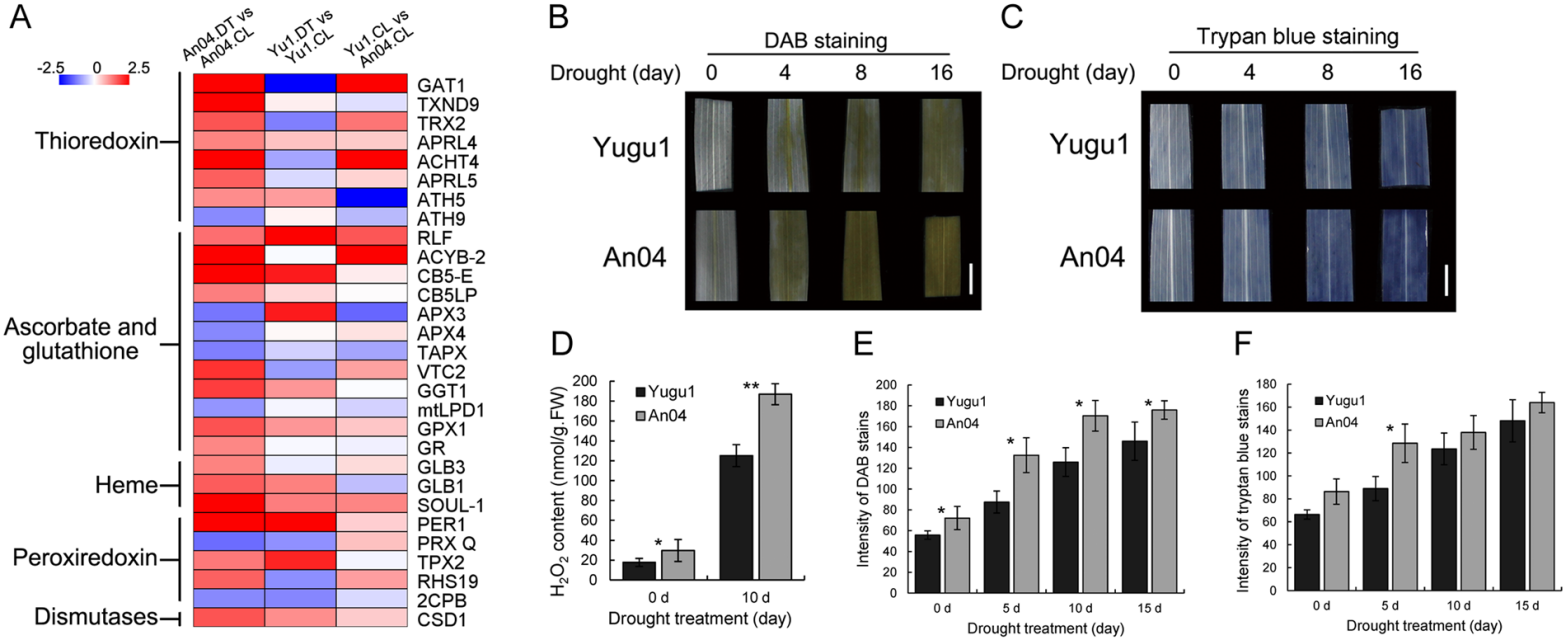
Regulation of reactive oxygen species (ROS) in ‘Yugu1’ and ‘An04’ genotypes. (**A**) Expression profiles of featured genes involved in ROS scavenging in ‘An04’ and ‘Yugu1’. (**B** and **C**) The histochemical staining analysis shows that the ROS accumulation (indicated by 3,3′-diaminobenzidine staining,) and cell death (indicated by Trypan blue staining) triggered by drought were more severe in drought-sensitive ‘An04’ than in ‘Yugu1’. (**D**) The content of H_2_O_2_ in the leaves extracts of ‘Yugu1’ and ‘An04’. Values are means ± SE of three independent experiments. (**E**,**F**) 3,3′-diaminobenzidine and Trypan blue stains were quantified with mean ± SE (three biological replications) using ImageJ software (https://imagej.net/). P values were calculated using Welch’s two-sample *t*-test (*P < 0.05, **P < 0.001).

In crops subjected to various abiotic stresses, including drought, the generation of ROS is an inevitable effect. Excessive ROS accumulation is toxic to plants and triggers cell death. In this study, 55 genes associated with the cellular detoxification process, including 21 genes encoding peroxiredoxin, 18 involved in the ascorbate and glutathione scavenging system, 9 encoding thioredoxin, and a few encoding superoxide dismutase, showed changes in expression levels in response to drought (Fig. 9A, Supplementary Table S11-detoxification). Of these, 39 (70.9%) were differentially expressed in the drought-sensitive variety, while less than 30% of them had altered expressions in ‘Yugu1’ which suggested that the drought-sensitive ‘An04’ may have large levels of ROS, while the drought-tolerant ‘Yugu1’ did not. DAB (3,3′-Diaminobenzidine) and Trypan blue staining of ‘An04’ and ‘Yugu1’ seedlings indicated that there were greater ROS accumulation and cell death levels in the drought-sensitive genotype (Fig. 9B–F). The changes in the production of H_2_O_2_ were further measured in *S. italica* leaf extracts. The result also supported that the drought-sensitive ‘An04’ accumulate a significantly greater level of H_2_O_2_ than ‘Yugu1’ under drought conditions (Fig. 9D). Thus, we concluded that the increased ROS concentration in ‘An04’ under drought stress may be correlated with the activation of ROS-scavenging genes and its drought-sensitive phenotype.

It is not surprising that a number of genes that participated in major and minor carbohydrate metabolism were abundant in our RNA-seq data because soluble carbohydrates can act as common osmoprotectants that enable plants to maintain cell turgor and water absorption under drought conditions. An osmotic adjustment was indicated by the significant up-regulation of most of the 43 DEGs involved in soluble carbohydrate synthesis, including raffinose and trehalose (Supplementary Table S11-carbohydrates). Similar regulation patterns have also been found in other drought-tolerant plant species^36^. Drought-induced soluble carbohydrate accumulation cannot be achieved solely by *de novo* synthesis but requires the hydrolysis of stored polysaccharides. In total, 26 genes involved in the degradation of the major carbohydrates were differentially expressed in drought-stressed *S. italica* (Supplementary Table S11-carbohydrates). Amylase is an enzyme that catalyzes the breakdown of starch into soluble sugars. In our study, the up-regulation of the *AMY1* and *BAM1*/*BMY3* genes in both drought-tolerant and -sensitive genotypes suggested their roles in the accumulation of starch-derived soluble carbohydrates in *S. italica* under drought stress.

Other drought-stress-related genes, including those for transporters, heat shock proteins (HSPs), LEAs, and universal stress proteins (USPs), are summarized in Supplementary Table S11-others. Aquaporins are integral membrane proteins that regulate water flow inside or outside of cells, and 13 aquaporins (5 PIPs, 5 TIPs and 3 NIPs) were observed to be differentially expressed in both *S. italica* varieties in response to drought. Additionally, 87 HSPs and 13 LEAs displayed differential accumulations during a water deficit, corroborating their protective roles as determined in various plants under abiotic stress^37, 38^. There were less gene expression changes in the abiotic-stress-related protective DEGs in ‘Yugu1’ than in ‘An04’, which implied that the drought-tolerant genotype had already acclimated to the drought environment, and its genes’ expression patterns were maintained relatively steadily during a water deficiency compared with those of ‘An04’.

### Identification of candidate genes that mapped to the previously identified drought-related QTLs in foxtail millet

On the basis of our previous work^15^, 12 QTLs controlling osmotic- or drought-tolerance-related traits in *Setaria* at germination and early seedling stages were selected in this study. Previous drought-related QTL mining was carried out using a RIL (recombinant inbred line) population derived from a cross between ‘Yugu1’, a *S. italica* cultivar, and ‘W53’, a wild *S. viridis* genotype. Although the drought-associated QTL mining was performed in *S. italica* and *S. viridis*, results from this experiment are comparable with the present study for two reasons: 1. *S. italica* is domesticated from its wild ancestor *S. viridis*, resulting in the two species having a very close phylogenetic relationship and genetic synteny; and 2. Their genes have almost identical physical positions, with the DNA sequences and functions being highly conserved between *S. viridis* and *S. italica* ^10, 39^. These QTLs are comparable and valuable for the present study. Thus, we integrated the QTL information with our RNA-seq data and *Setaria* tissue-specific gene expression data^40^. A comprehensive analysis helped to identify 20 candidate genes that may contribute to germination and early seedling’ drought tolerance in *Setaria* (Table 1). The information may also be useful for designing molecular markers for drought-tolerant plant breeding.

**Table 1:**
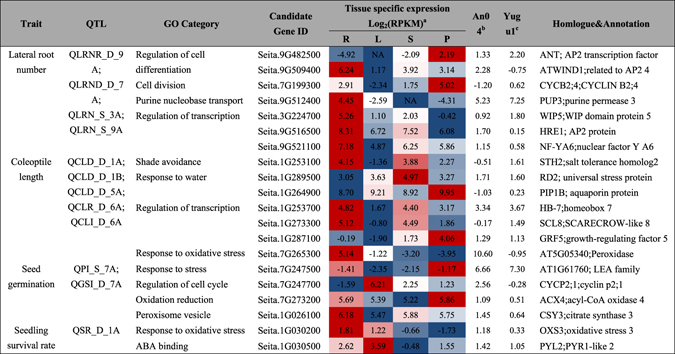
Identification of candidate genes localized within the QTLs associated with seedling drought tolerance in *Setaria*. ^a^R, L, S, and P represent root, leaf, stem, and panicle, respectively; ^b^Expression value of Log_2_ (An04.DT *vs* An04.CL); ^c^Expression value of Log_2_ (Yu1.DT *vs* Yu1.CL). The color gradient indicates the gene expression levels in different tissues.

The four QTLs (QLRNR_D_9 A, QLRND_D_7 A, QLRN_S_3 A, and QLRN_S_9 A) are important for controlling the lateral root number in *Setaria* seedlings in response to drought. In total, the 108 DEGs localized within these four QTL intervals were induced by drought in either ‘Yugu1’ or ‘An04’. A GO enrichment analysis showed that genes involved in TF activity (GO:0045449) and the regulation of cell differentiation (GO:0045595) were significantly enriched. Tissue-specific gene expression profiles showed that 24 DEGs had higher expression levels in roots than other tissues. Among them, three genes that may contribute to the maintenance of cell division and cytokinin transportation under drought conditions were of particular interest. *Seita.7G199300* belongs to a core cell cycle gene family (*CYCD*), and *Seita.9G482500* is homologous to a putative transcriptional regulator AINTEGUMENTA (*ANT*). In *Arabidopsis*, *ANT* is required for the control of cell proliferation. Randall *et al*. (2015) suggested that *ANT* and the D-type cyclin *CYCD3;1* are expressed in roots, respond to cytokinins, and are both required for secondary root development^41^. *Seita.9G512400* encodes a purine transporter. It was extremely highly expressed in *S. italica* roots, and responded to drought stress in ‘Yugu1’. Its homologous gene, *AtPUP3*, mediates cytokinin transportation across the plasma membrane^42^. We also found four TFs within the mapping interval, one of which, *Seita.9G509400* (homologue to *AtWIND1*, a member of the *DREB* subfamily), has been reported to be a key molecular switch that controls cell differentiation in *Arabidopsis* ^43^.

Using the same pipeline, we also identified four candidate genes in two major QTLs (QPI_S_7 A and QGSI_D_7 A) related to seed germination, seven genes related to coleoptile development were found in five QTLs (QCLD_D_1 A, QCLD_D_1B, QCLD_D_5 A, QCLR_D_6 A and QCLI_D_6 A), and two genes related to the seedling survival rate were found in one QTL (QSR_D_1 A) under drought stress. GO annotations of “response to water” and “response to oxidative stress” were enriched for DEGs located in these mapping intervals, indicating that these were important biological processes that may be responsible for germination and early seedling’ drought tolerance in *S. italica*. An analysis of the homologies of these candidate genes based on previously known pathways revealed that some genes controlled related traits in other model plants. For example, *Seita.1G030500*, which encodes a PYL/RCAR family protein, was identified as associating with the seedling survival rate in *S. italica*, and it was characterized as a regulatory component of the ABA receptor in *Arabidopsis* ^22^. The rice homologue *OsPYL* (Os02g13330) is a positive regulator of ABA signaling in seed germination and early seedling growth^23^. *STH2* (*Seita.1G253100*) is one of the seven genes that contribute to coleoptile length under drought stress. The gene was highly expressed in stems and roots, and was especially up-regulated by drought in ‘Yugu1’. In *Arabidopsis*, *STH2* is involved in light-dependent development and hypocotyl elongation^44^. When better characterized, these genes may provide information useful for understanding the drought adaptation of crops.

## Materials and Methods

### Plant materials, drought treatment, and physiological tests

Two cultivars of foxtail millet with contrasting sensitivities to drought stress, ‘Yugu1’ (drought-tolerant) and ‘An04’ (drought-sensitive), were employed in this study. Mature non-dormant seeds were germinated on moist filter paper in covered petri dishes for 16 h and then transplanted into individual pots (15-cm diameter, 35-cm depth) containing nutrient soil and vermiculite (1:1, v/v). The plants were placed in an experimental greenhouse at the Chinese Academy of Agricultural Sciences (Beijing, China) and grown for 3 weeks under controlled environmental conditions of 30 °C day/22 °C night with a 16 h light/8 h dark photoperiod prior to beginning the experiments. For the drought-stress treatments, 40 uniformly developed seedlings (approximately 4–5-cm height, with four leaves) were selected and divided into four groups. Control groups, Yugu1.CL and An04.CL, consisted of ‘Yugu1’ and ‘An04’ plants, respectively, that were watered to field capacity every day, while drought-treated groups, Yugu1.DT and An04.DT, contained seedlings of ‘Yugu1’ and ‘An04’, respectively, from which water was withheld until the soil’s gravimetric water content reached 21.5% of field capacity (Fig. 1B). At that point, the pre-dawn leaf water potential of ‘An04’ seedlings decreased by approximately one-half in the drought treatment group compared with in the well-watered group (Fig. 1C). Each group contained at least 20 independent seedlings that could be used for physiological measurements.

Physiological measurements, including LWP, Fv/Fm, ΦPSII, photosynthetic electron transport rate (ETR), and qP, were taken nondestructively throughout the treatment periods. The soil volumetric water content was measured using a Field Scout^™^ TDR 300 Soil Moisture Meter (Spectrum Technologies, Canada). The LWP was measured using the PSYPRO Water Potential System (WESCOR Inc., USA). ΦPSII, ETR, qP, and Fv/Fm were measured using the Li-6400 platform (LI-COR Inc., USA). Each group included measurements for 18 biological replications. Parameters used in physiological measurements were set based on our previous report^36^.

At the mature stage, 10 uniformly-developed plants of both ‘Yugu1’ and ‘An04’ were collected for agronomic trait investigation. Major agronomic traits, including plant height, panicle length and 1,000-grain weight, were measured in 10 biological replications. The agronomic traits’ investigation and scoring standards were described in our previous study^14^.

### RNA-seq library construction and sequencing

After the drought treatment, the aboveground parts of seedlings were collected from the control and drought-treated groups, with three independent biological replications for each group, frozen in liquid nitrogen, and stored at −80 °C for total RNA isolation and cDNA library construction. Total RNA was extracted by the standard TRIzol method (Ambion, Thermo Scientific Inc., USA). The cDNA libraries of Yugu1.CL, An04.CL, Yugu1.DT, and An04.DT were constructed and sequenced on Illumina HiSeq. 2000 platform in 100-bp paired-end mode (Illumina Inc., USA), with three independent biological replications for each sample. Sequencing data obtained in this study have been deposited at EMBL-EBI (http://www.ebi.ac.uk/) under accession number PRJEB21225.

### Analysis of the transcriptomic data and qPCR validation

After screening and trimming, clean sequencing reads were aligned to the reference *S. italica* genome V2.2 (phytozome.jgi.doe.gov) using TopHat2^45^. Gene expression was estimated using three different packages, Cufflinks^19^, EdgR^20^, and DESeq. 2^21^. Genes confirmed by at least two computational methods with estimated absolute fold changes ≥ 1 and false discovery rates ≤ 0.01, were identified as reliable DEGs. Functional enrichment analyses of these DEGs were performed using MapMan^46^, Bingo^47^, and Kobas^48^. The transcriptomic analysis results were visualized using R (https://www.r-project.org/) and Cytoscape software^49^. Seventeen DEGs were randomly selected to validate the gene expression in the Illumina data using qRT-PCR as described previously^50^. The primers and qRT-PCR results are listed in Supplementary Table S2.

### Endogenous phytohormone quantification and histochemical staining analysis

To test the induction of phytohormones in ‘Yugu1’ and ‘An04’ in response to drought, foxtail millet seedlings were subjected to a natural water withholding. Endogenous ABA, GA, auxin, BR, and ZR (an active cytokinin in plants) levels in the uppermost leaves were quantified using the method reported by Yang *et al*.^51^. The raw data of endogenous phytohormone measurements are provided in Supplementary Table S12. Trypan blue staining, which is used to visualize dying cells, and DAB staining, which can detect ROS accumulation, were performed on drought-stressed foxtail millet seedlings as described previously^52^. Quantification of the staining was performed using ImageJ software (https://imagej.net/). The production of H_2_O_2_ in *S. italica* leaf extracts was measured using a Hydrogen Peroxide Assay Kit (Beyotime Institute of Biotechnology, Shanghai, China) as previously reported^53^.

## Conclusions

In recent years, the ability of crops to maintain normal growth and increase their water use efficiency when the water supply is insufficient has been determined to be more valuable than engineering crops that can survive under extreme drought conditions. *S. italica* is a crop that can maintain normal growth in arid regions. Our study presents phenotypic and transcriptomic differences between two *Setaria* varieties that have different sensitivities to drought. ‘An04’ was identified as a drought-sensitive genotype, and ‘Yugu1’ was identified as more drought-tolerant. By linking the drought-associated transcriptome to physiological responses in the two genotypes, it was determined that ‘Yugu1’ could repress the energy metabolism to protect plant cells and maintain growth under drought conditions. However, ‘An04’ consumed more energy, and its photosynthesis efficiencies and cell growth were severely inhibited under drought conditions. Gene expression profiling and a cluster analysis suggested that transcriptomic regulatory mechanisms controlling stress responses in *S. italica* were greatly affected by genotype × environment interactions, which are thus crucial for determining the stress responses and transcriptomic compositions of different genotypes. A functional enrichment analysis indicated that genes involved in phytohormone metabolism and signalling, TFs, cellular detoxification, and osmotic adjustment may play important roles in modulating the genotype-specific drought stress responses of *S. italica*. After combining the RNA-seq data with our previously mapped QTLs, 20 potential candidate genes were identified. Foxtail millet had a high percentage of syntenic relationships at the genomic level to main Poaceae crops. Thus, information acquired in this study will not only increase the understanding of drought mechanisms in millet crops but will also be valuable for generating elite cultivars of other major cereals having durable drought tolerances. Further research using transgenic engineering is needed to validate the functions of these candidate genes, and molecular markers designed for the validation could be used for drought-tolerant crop improvement.

### Data availability

Sequencing data have been deposited at EMBL-EBI (http://www.ebi.ac.uk/) under accession number PRJEB21225.

## Electronic supplementary material

Supplementary Information

Dataset 1

Dataset 2

Dataset 3

Dataset 4

Dataset 5

Dataset 6

Dataset 7

Dataset 8

Dataset 9

Dataset 10

Dataset 11

Dataset 12
