# Supplementary material for: Genotype-specific physiological and transcriptomic responses to drought stress in Setaria italica (an emerging model for Panicoideae grasses)
Source: Sci Rep. 2017 Aug 30;7:10009. doi: 10.1038/s41598-017-08854-6 (PMC5577110; doi:10.1038/s41598-017-08854-6)
Supplement: Supplementary file 1 — Supplementary Information [file 41598_2017_8854_MOESM1_ESM.pdf]

Genotype-specific physiological and transcriptomic responses to drought stress in *Setaria italica* (an emerging model for Panicoideae grasses)

Sha Tang[1, a], Lin Li[2, a], Yongqiang Wang[3, a], Qiannan Chen[2], Wenying Zhang[4], Guanqing Jia[1], Hui Zhi[1], Baohua Zhao[2], Xianmin Diao[1, 2, *]

1. Institute of Crop Sciences, Chinese Academy of Agricultural Sciences, 100081 Beijing, People's Republic of China

2. College of Life Science, Hebei Normal University, 050012 Shijiazhuang, People's Republic of China

3. Institute of Cotton, Hebei Academy of Agricultural and Forestry Sciences, 05003, Shijiazhuang, People's Republic of China

4.Institute of Dryland Agriculture, Hebei Academy of Agricultural and Forestry Sciences, 050000, Hengshui, People's Republic of China

[a] These authors contributed equally to this work.

[*] Corresponding Author: Xianmin Diao

Address: Institute of Crop Sciences, Chinese Academy of Agriculture Sciences, No.12 Zhongguancun South Street, Haidian, Beijing, 100081, P. R. China.

Phone: (86)1062126889; Fax: (86)1062126889

Email: diaoxianmin@caas.cn

Supplementary Information

Supplementary Table S1. Chlorophyll fluorescence measurement for *S. italica* subjected to water stress treatment.

Supplementary Table S2. qRT-PCR validation of differentially expressed genes.

Supplementary Table S3. Correlation between qRT-PCR and RNA-Seq data.

Supplementary Table S4. Expression profile and gene annotation of differential expressed genes in RNA-seq.

Supplementary Table S5. GO enrichment analysis of differentially expressed genes in each of six K-means clusters.

Supplementary Table S6. KEGG enrichment analysis of differentially expressed genes in each of six K-means clusters.

Supplementary Table S7. Comparison of major agronomic traits between the 'An04' and 'Yugu1'.

Supplementary Table S8. GO enrichment analysis of genes in different category of Figure 6A.

Supplementary Table S9. Differential expressed genes involved in phytohormone metabolism and signaling.

Supplementary Table S10. List of differentially expressed transcription factors.

Supplementary Table S11. List of differentially expressed protective genes involved in drought responses in *S. italica*.

Supplementary Table S12. Quantification of endogenous phytohormones in 'Yugu1' and 'An04' in response to drought.

Supplementary information is available on the website. Please see the online Excel files for details.
